# Multifunctional Nanocarriers Based on Chitosan Oligomers and Graphitic Carbon Nitride Assembly

**DOI:** 10.3390/ma15248981

**Published:** 2022-12-15

**Authors:** Alberto Santiago-Aliste, Eva Sánchez-Hernández, Natalia Langa-Lomba, Vicente González-García, José Casanova-Gascón, Jesús Martín-Gil, Pablo Martín-Ramos

**Affiliations:** 1 Department of Agricultural and Forestry Engineering, ETSIIAA, University of Valladolid, Avenida de Madrid 44, 34004 Palencia, Spain; 2 Instituto Universitario de Investigación en Ciencias Ambientales de Aragón (IUCA), EPS, University of Zaragoza, Carretera de Cuarte s/n, 22071 Huesca, Spain; 3 Plant Protection Unit, Instituto Agroalimentario de Aragón-IA2 (CITA-Universidad de Zaragoza), Avda. Montañana 930, 50059 Zaragoza, Spain

**Keywords:** chitosan oligomers, cross-linking, g-C_3_N_4_, grapevine phytopathogens, integrated pest management, methacrylic anhydride, photocatalytic degradation, photocatalytic reduction

## Abstract

In this study, a graphitic carbon nitride and chitosan oligomers (g-C_3_N_4_–COS) nanocarrier assembly, which was obtained by cross-linking with methacrylic anhydride (MA), was synthesized and characterized. Its characterization was carried out using infrared spectroscopy, elemental and thermal analyses, and transmission electron microscopy. The new nanocarriers (NCs), with an average particle size of 85 nm in diameter and a 0.25 dispersity index, showed photocatalytic activity (associated with the g-C_3_N_4_ moiety), susceptibility to enzymatic degradation (due to the presence of the COS moiety), and high encapsulation and moderate-high release efficiencies (>95% and >74%, respectively). As a proof of concept, the visible-light-driven photocatalytic activity of the NCs was tested for rhodamine B degradation and the reduction of uranium(VI) to uranium(IV). Regarding the potential of the nanocarriers for the encapsulation and delivery of bioactive products for crop protection, NCs loaded with *Rubia tinctorum* extracts were investigated in vitro against three *Vitis vinifera* phytopathogens (viz. *Neofusicoccum parvum*, *Diplodia seriata*, and *Xylophilus ampelinus*), obtaining minimum inhibitory concentration values of 750, 250, and 187.5 µg·mL^−1^, respectively. Their antifungal activity was further tested in vivo as a pruning wound protection product in young ‘Tempranillo’ grapevine plants that were artificially infected with the two aforementioned species of the family *Botryosphaeriaceae*, finding a significant reduction of the necrosis lengths in the inner woody tissues. Therefore, g-C_3_N_4_-MA-COS NCs may be put forward as a multifunctional platform for environmental and agrochemical delivery applications.

## 1. Introduction

Engineered nanocarriers (NCs) have substantially contributed to the development of precision medicine [1], and, more recently, they are also finding applications in agriculture as a promising route to increase crop production while reducing the environmental impact associated with food production and crop protection [2].

For agricultural purposes, the main characteristic of any nanocarrier is to exhibit controlled release properties and site-specific delivery, i.e., the release of the active ingredient should respond to the stimuli produced by the pest and/or the surrounding environment [3]. Nonetheless, other features are also highly desirable: good physical and chemical properties, stability in different media, a high adsorption capacity, ease of modification to tune the surface characteristics such as charge and permeability, biocompatibility, biodegradability, low toxicity, and competitive production costs [2].

The state-of-the-art of micro- and nanocarriers used for the encapsulation of agrochemicals have been covered in several recent review papers [3,4], which showed that naturally sourced polymers, such as chitosan (36%), alginate (23%), and plant-based proteins (16%); synthetic polymers (35%); and inorganic materials (e.g., metal-organic frameworks and calcium carbonate) are the among the options that have been explored for the construction of NCs. 

Concerning the preferred option (viz. chitosan), the agronanochemicals for crop protection based on this natural polymer would feature several attractive properties, as discussed in the review by Maluin and Hussein [5]. The encapsulation of the active ingredients in chitosan NCs shields the toxic effect of the free agrochemicals on the plant and minimizes the negative impacts on the environment and human health, it increases the uptake due to the enhanced penetration of the nanometer-sized particles on the plant cell wall and cuticle, and it minimizes the wastage and leaching of the active ingredients due to the controlled release properties and high bioavailability of the nanoformulations. Further, chitosan features amphiphilic and bioadhesive properties, enhances solubility and stability, and its use can result in synergistic effects. Chitosan may also be used for nutrient encapsulation, i.e., for the preparation of nanofertilizers [6], and it elicits immune-modulatory activity. In this regard, it should be clarified that chitin has a pathogen-associated molecular pattern, which can be detected by the LysM/CERK1 transmembrane chitin receptor in the plant cells, and its sensing triggers an intracellular defense immune response (involving the activation of kinases and up-regulation of defense-related genes, such as plant defensin PDF1.2) [2]. For environmental remediation purposes, chitosan can be used as a flocculant and coagulant, and as an adsorbent for the removal of pollutants such as heavy metals, pesticides, dyes, antibiotics, and biological contaminants from wastewater [7].

The development of cross-linked chitosan-based systems for drug delivery usually relies on tripolyphosphate (TPP), glutaraldehyde (GLA), ethylene glycol diglycidyl ether, epichlorohydrin, polyethyleneimine, and phenylalanine chemical cross-linkers [8], although some of them (e.g., GLA) are considered to be toxic and can inactivate macromolecular drugs [9], while the others (e.g., TPP) face aggregation and dissolution stability problems [10]. The aforementioned issues can be overcome by using anhydride methacrylate (MA) as an alternative cross-linker. MA has been commonly used to modify the chemical structure of various natural polymers and biomolecules—including polysaccharides—with a significant coupling efficiency under mild reaction conditions [11], without affecting the biocompatibility of the final product [12]. The carbon–carbon double bonds of the methacryl groups can react with hydroxyl- and amino-groups, leading to the covalent attachment of methacryloyl moieties, which can be used in subsequent cross-linkage reactions. Modification with MA has been reported to improve the encapsulation of the bioactive compounds [13] and to enhance the water solubility and the capacity of the gel and scaffold formation of chitosan [14]. Concerning NCs preparation, MA has been successfully used as a cross-linking agent in lignin–bio-based amine NCs [15] and lignin–chitosan oligomers-based NCs [16]. 

To exploit the susceptibility of MA to act as a link between the related chemical species, resulting in a ternary complex with transporter properties, and to broaden the profile of the analogous complexes, in this study, we have explored the feasibility of the synthesis and behavior of the graphene carbon nitride-methacrylate-chitosan oligomers (g-C_3_N_4_-MA-COS) system as a nanocarrier. 

Regarding the inclusion of the g-C_3_N_4_ moiety in the g-C_3_N_4_-MA-COS assembly, it should be noted that carbon nitride has gained extensive attention due to its excellent physicochemical properties, attractive electronic band structure, and low cost. Concerning the functions enabled by g-C_3_N_4_ toward the end applications of the multifunctional NCs, g-C_3_N_4_ nanosheets have been shown to be a suitable functional component for bioactive product delivery due to their low toxicity, excellent biocompatibility, high penetration into tissues ability, photosensitive and pH-sensitive properties, efficiency in drug encapsulation (associated with their large surface area), and positive effect on the release of encapsulated compounds [17]. Regarding the environmental applications, g-C_3_N_4_-based photocatalysts are very efficient for pollutant degradation and bacterial disinfection [18,19].

The use of g-C_3_N_4_ in combination with chitosan has precedents in, for instance, the work by Gupta and Gupta [20] on chitosan hydrogels that were embedded with g-C_3_N_4_/ZnO nanoparticles for ciprofloxacin removal or in the chitosan films incorporated with curcumin-loaded hollow g-C_3_N_4_ nanoparticles (prepared using nanosized silica template) for bananas preservation that was reported by Ni et al. [21]. pH-sensitive NCs based on a composite of chitosan/agarose/g-C_3_N_4_ were also investigated by Rajabzadeh-Khosroshahi et al. [17] as a drug delivery system for anticancer curcumin release, in which glyoxal was used to cross-link chitosan and agarose (2:1, *w/w*). Likewise, a system based on Pd nanoparticles embedded in micro-sized chitosan-g-C_3_N_4_ hybrid spheres (0.9 mm in diameter) was reported by Yılmaz Baran et al. [22] for the treatment of environmental pollutants in the aqueous medium, although in this case the chitosan-g-C_3_N_4_ mixture spheres were obtained through a spherification process of chitosan (using GLA), in which g-C_3_N_4_ did not intervene. Hence, to the best of the authors’ knowledge, the actual cross-linkage between chitosan and g-C_3_N_4_ to form NCs has not been explored to date.

In the work presented herein, we report the synthesis and characterization of multifunctional NCs based on the chemical cross-linkage of COS and g-C_3_N_4_ using MA. To assess the presumed versatility of these g-C_3_N_4_-MA-COS NCs, environmental remediation (with two paradigmatic pollutants, rhodamine B dye and uranium(VI)), and biorational-based antimicrobial delivery applications in agriculture (against three emerging grapevine pathogens) were also investigated.

## 2. Material and Methods

### 2.1. Reagents

High molecular weight chitosan (CAS No. 9012-76-4; MW: 310,000–375,000 Da) was supplied by Hangzhou Simit Chem. & Tech. Co. (Hangzhou, China). Melamine cyanurate (CAS No. 37640-57-6; >99.0%) was purchased from Nachmann S.r.l. (Milano, Italy). Neutrase^TM^ 0.8 L enzyme was supplied by Novozymes A/S (Bagsværd, Denmark). Chitosanase from *Streptomyces griseus* (Krainsky) Waksman and Henrici (EC 3.2.1.132, CAS No. 51570-20-8), acetic acid (purum, 80% in H_2_O; CAS No. 64-19-7), rhodamine B (analytical standard, CAS No. 81-88-9), methacrylic anhydride (CAS No. 760-93-0; ≥94%), Arsenazo III (CAS No. 1668-00-4), methanol (UHPLC, suitable for mass spectrometry, CAS 67-56-1), tetrahydrofuran (THF, CAS No. 109-99-9; ≥99.9%), tryptic soy agar (TSA, CAS No. 91079-40-2), and tryptic soy broth (TSB, CAS No. 8013-01-2) were supplied by Sigma–Aldrich Química (Madrid, Spain). Uranyl nitrate hexahydrate (CAS 13520-83-7, ACS grade, Honeywell Fluka) was supplied by Fisher Scientific SL (Madrid, Spain). Potato dextrose agar (PDA) was purchased from Becton Dickinson (Bergen County, NJ, USA). 

### 2.2. Fungal and Bacterial Isolates

The two fungal isolates under study, *Neofusicoccum parvum* (Pennycook and Samuels) Crous, Slippers, and Phillips (code ITACYL F111, isolate Y-091-03-01c) and *Diplodia seriata* de Notaris (code ITACYL F098, isolate Y-084-01-01a) were kindly supplied by the Instituto Tecnológico Agrario de Castilla y León (ITACYL, Valladolid, Spain) as lyophilized vials, which were later reconstituted and refreshed as PDA subcultures. Regarding the bacterial isolate, *Xylophilus ampelinus* (Panagopoulos) Willems, Gillis, Kersters, van den Broeke, and De Ley was acquired by the Spanish Type Culture Collection (CECT), with a CCUG 21,976 strain designation.

### 2.3. Chitosan Oligomers and g-C_3_N_4_ Preparation

The chitosan oligomers (COS) were prepared according to the procedure described in the work by Santos-Moriano et al. [23] with the modifications indicated in [16] using the commercial proteolytic preparation Neutrase^TM^ to degrade the chitosan polymer chains and obtain a product enriched in deacetylated chitooligosaccharides. At the end of the process, a solution with a pH in the range from 4 to 6 with oligomers of a molecular weight of <2 kDa was obtained, with a polydispersity index of 1.6, which is within the usual range that has been reported in the literature [24].

High-purity nanosheets of g-C_3_N_4_ were obtained from the pyrolysis of melamine cyanurate in a capped crucible under an air atmosphere at 600 °C for 50 min according to the procedure previously reported by our group in [25].

### 2.4. Plant Material and Preparation of Extract

*Rubia tinctorum* L. specimens used for the preparation of the bioactive extract to be encapsulated were collected from the banks of the Carrión River as it passes through the town of Palencia (Spain). The details of the hydromethanolic extract preparation, its characterization using gas chromatography-mass spectroscopy (GC-MS), and its antimicrobial activity have been previously reported in [26].

‘Tempranillo’ grapevine plants used in the in vivo study were supplied by VCR Vivai Cooperativi Rauscedo (Italy), with supplier ID IT-06-1031. The clone was ‘CL. 32’ and the rootstock was ‘775P CFC 83/20’. The lot number was ‘PN 001 19/1519507’.

### 2.5. Synthesis of the g-C_3_N_4_-MA-COS Nanocarriers

The preparation of the nanocarriers was carried out as described in patent P202230668 [27]. The synthesis of methacrylated chitosan was conducted according to the procedure proposed by Gupta and Gupta [20], but with modifications. In brief, the methacrylation of COS was performed by the addition of 420 mg of oligomers which were dispersed in a solution of methacrylic anhydride in tetrahydrofuran (THF), which was obtained by dispersing 0.5 mL of MA (*ρ* = 1.035 g·cm^−3^) in 25 mL of THF. The mixture was sonicated for 5 min (distributed in 1 min periods) using a probe-type ultrasonicator (model UIP1000hdT; 1000 W, 20 kHz; Hielscher Ultrasonics, Teltow, Germany). The co-encapsulating chemical species was a porous form of g-C_3_N_4_ resulting from the attack of 210 mg of g-C_3_N_4_ with MA in THF (0.5 mL in 25 mL). The methacrylated g-C_3_N_4_ solution was then added dropwise to the methacrylated COS solution, which was followed by sonication for 5 min (distributed in 1 min periods) to obtain a g-C_3_N_4_:COS weight ratio of 0.5:1 (with an unknown MA proportion). It should be clarified that the other assayed weight ratios did not result in the formation of NCs or lead to non-monodisperse size distributions. The excess MA was removed by agitation and successive washings.

### 2.6. Encapsulation and Release of R. tinctorum Extract

For the agrochemical delivery tests, *R. tinctorum* extract was chosen as an example of a bioactive agent because it has previously shown high inhibitory efficacy against the aforementioned fungal and bacterial phytopathogens, which were both unencapsulated [26] and encapsulated in lignin–chitosan nanocarriers [16].

To prepare the nanocarriers with the encapsulated *R. tinctorum* extract, 105 mg of the lyophilized extract that was to be encapsulated was added to the g-C_3_N_4_-MA-COS solution to obtain a g-C_3_N_4_:COS:*R. tinctorum* 0.5:1:0.25 weight ratio. The mixture was subjected to sonication for 1 h, distributed in 5 min periods, while the temperature (which was always lower than 60 °C) and the pH (4-5) were controlled.

Concerning the plant extract encapsulation efficiency (EE), it was determined using the indirect method that was proposed by Fischer et al. [28]; the sample was centrifuged at 10,000 rpm (60 min), and the supernatant containing the non-encapsulated plant extract was first freeze-dried, then redissolved in methanol:water (1:1, *v/v*), passed through a 0.2 μm filter, and analyzed by high-pressure liquid chromatography (HPLC) using an Agilent 1200 series HPLC system (Agilent Technologies, Santa Clara, CA, USA). The operative conditions were [29]: methanol/5% acetic acid (pH 3) (70~30) mobile phase; 10 μL injection volume; 20 °C column temperature; 0.2 mL·min^−1^ flow rate; G1315D detector operated at 250 nm. The encapsulation efficiency was determined as $\mathrm{EE}\left(\%\right)=\left(m\left(\mathrm{extract}\;\mathrm{initial}\right)-m\left(\mathrm{extract}\;\mathrm{supernatant}\right)\right)/m\left(\mathrm{extract}\;\mathrm{initial}\right)\times 100$.

As for the release efficiency (RE), the release assays were performed by the addition of a weighted quantity of freeze-dried loaded NCs (obtained from the encapsulation efficiency test) and 2.5 U of chitosanase (EC 3.2.1.132) to a methanol:water (1:1, *v/v*) solution under light stirring (150 rpm) in the dark for 2 h. An aliquot was sampled, and the released *R. tinctorum* extract was assayed by the same method that was employed for the determination of residual (i.e., non-encapsulated) extract. The release efficiency was calculated from the amount of extract released as a percentage of the total amount of extract encapsulated in the NCs.

### 2.7. Characterization

The multi-elemental composition of the NCs, before and after encapsulation of *R. tinctorum* extract, was analyzed by scanning electron microscopy with energy-dispersive X-ray spectroscopy (SEM-EDX) using an EVO HD 25 (Carl Zeiss, Oberkochen, Germany) apparatus.

Infrared vibrational spectra were collected using a Thermo Scientific (Waltham, MA, USA) Nicolet iS50 FTIR spectrometer equipped with an integrated diamond attenuated total reflection (ATR) system. The spectra have been recorded with a spectral resolution of 1 cm^−1^ in the range of 400–4000 cm^−1^, taking the interferograms resulting from the co-addition of 64 scans.

The Transmission Electron Microscopy (TEM) characterization was performed using a JEOL (Akishima, Tokyo, Japan) JEM 1011 HR microscope. The operating conditions were: 100 kV; 25,000–120,000× magnification. Micrographs were obtained using a GATAN ES1000W CCD camera (4000 × 2672 pixels). Uranyl acetate (2%) was used for the negative staining of the samples. 

The dispersity was calculated from the TEM data as $p=\sigma /R_{avg}$, where *p* is the dispersity, *σ* is the standard deviation of a radius in a batch of NCs, and *R*_avg_ is the average radius of the NCs [30]. 

The thermogravimetric/derivative thermogravimetric (TG/DTG) analyses were conducted using a TG-DSC2 (Mettler-Toledo, Columbus, OH, USA) thermal analyzer by heating the sample in a slow stream of N_2_ (20 mL·min^−1^) from room temperature up to 750 °C at a heating rate of 20 °C·min^−1^.

### 2.8. Photocatalytic Activity

To evaluate the photocatalytic activity of the g-C_3_N_4_-MA-COS NCs, rhodamine B (RhB) dye degradation tests were carried out under visible light irradiation (λ > 420 nm) following the protocol described by Dong and Zhang [31], but with minor modifications. A 300 W xenon lamp with a 420 nm cutoff filter was chosen as a visible light source. The nanocarriers (0.1 g) were dispersed into 100 mL of 10 mg·L^−1^ RhB aqueous solution in a container with a cooling water jacket that was on the outside. To obtain an adsorption–desorption equilibrium between the assayed material and RhB, the solution was stirred in the dark for 2 h. During the irradiation, about 4 mL of the suspensions were taken from the reaction cell at 10 min intervals (over a 2 h period), and then, they were centrifuged to remove the nanoparticles. The RhB concentration was determined by taking absorbance measurements at 550 nm using a Multiskan GO Microplate spectrophotometer (Fisher Scientific). The reaction constant was calculated as $\ln\left(C/C_{0}\right)=-kt$, where *C* is the maximum peak of the absorption spectra of RhB for each irradiated time interval, and *C*_0_ is the absorption of the starting concentration when the adsorption/desorption equilibrium was achieved.

Additional photocatalytic tests were conducted to investigate the ability of the NCs to reduce U(VI) to U(IV) under visible light following the methodology that is described in [32], but with modifications. In the catalytic process, 30 mg of g-C_3_N_4_-MA-COS NCs was added into 60 mL of 3 mg·L^−1^ UO_2_(NO_3_)_2_ solution (containing 3 mL methanol as the electron sacrifice). The pH value was adjusted to 6.0. As in the RhB procedure described above, the visible light irradiation was obtained using a 300 W Xe lamp equipped with a 420 nm cutoff filter. Before irradiation, the reaction system was bubbled with N_2_ in the dark for 2 h to reach the adsorption–desorption equilibrium and maintain the anaerobic conditions. After irradiation, 1 mL of suspension was pipetted out at a certain time and rapidly filtered. The U(VI) concentration was measured by UV-vis spectrophotometry at 652 nm using Arsenazo III. The photoreaction rate constant was calculated based on pseudo-first-order kinetics as $\ln\left(C/C_{0}\right)=-kt$, where *C* represents the concentration of uranium(VI) at a given time, and *C*_0_ represents the initial concentration.

### 2.9. In Vitro Antimicrobial Activity

The g-C_3_N_4_-MA-COS nanocarriers, before and after encapsulation of *R. tinctorum* extract, were first assayed against *X. ampelinus*. The antibacterial activity was evaluated by determining the minimum inhibitory concentration (MIC). The agar dilution method was used according to CLSI standard M07-11 [33]. An isolated colony of *X. ampelinus* was cultured in TSB liquid medium at 26 °C for 18 h. Starting from a concentration of 10^8^ CFU·mL^−1^, serial dilutions were made to obtain a final inoculum of ~10^4^ CFU·mL^−1^. Subsequently, the bacterial suspension was applied to the surface of tryptic soy agar (TSA) plates that were amended with the treatments at concentrations ranging from 62.5 to 1500 μg·mL^−1^. The plates were incubated at 26 °C for 24 h. The MICs were determined as the lowest concentrations at which no bacterial growth was observed in the agar dilutions. All of the experiments were performed in triplicate, and each replicate consisted of three plates per treatment/concentration.

Regarding the inhibition of mycelial growth, which was tested against *D. seriata* and *N. parvum*, it was determined by dilution in agar, according to the EUCAST antifungal susceptibility testing standard procedures [34], incorporating aliquots of stock solutions (of NCs, either empty or loaded with *R. tinctorum*) onto a potato dextrose agar (PDA) medium to obtain concentrations in the range of 62.5–1500 μg·mL^−1^. Fungal mycelium plugs (⌀ = 5 mm) were transferred from the margins of one-week-old *D. seriata* or *N. parvum* PDA cultures to plates incorporating the concentrations mentioned for each treatment (three plates per treatment/concentration with two replicates each). The plates were then incubated at 25 °C in the dark for one week. A PDA medium without any modification was used as control. The mycelial growth inhibition was estimated according to the formula: ((*d_c_* − *d_t_*)/*d_c_*) × 100, where *d_c_* and *d_t_* represent the mean diameters of the control fungal colony and the treated fungal colony, respectively. The effective concentrations (EC_50_ and EC_90_) were estimated using the PROBIT analysis in IBM SPSS Statistics v.25 (IBM; Armonk, NY, USA).

### 2.10. In Planta Bioassays

To determine the in vivo protective activity, bioassays were carried out with the g-C_3_N_4_-MA-COS NCs loaded with the hydromethanolic extract of *R. tinctorum* in 2-year-old ‘Tempranillo’ grapevine plants that were artificially infected with the two selected Botryosphaeriaceous fungi. Each plant was grown in a 3.5 L plastic pot with a mixed substrate of peat and sterilized natural soil (75:25), incorporating slow-release fertilizer when it was necessary throughout the study period. One week after potting, the young grafted plants were ‘wounded’ on the trunk at two sites per stem (separated >5 cm), which were below the grafting point and without reaching the root crown. Thus, slits of approx. 15 mm diameter and 5 mm deep were made using a scalpel. The g-C_3_N_4_-MA-COS NCs loaded with a hydromethanolic extract of *R. tinctorum* (2 mL, at concentrations of 250 and 750 μg·mL^−1^ against *D. seriata* and *N. parvum*, respectively) were then applied to each of the wounds using a pipette, and it was allowed to dry. Subsequently, a 5 mm diameter agar plug coming from the margin of a fresh 5day-old PDA culture of the fungal species (either *D. seriata* or *N. parvum*) was placed directly in contact with the vascular tissue in the stem at each wound, and the wound was covered with sterile cotton soaked in sterile bi-distilled water and sealed with Parafilm^TM^ tape. Twenty-eight replicates (plants) were set up for each pathogen, along with four positive controls per pathogen, plus four negative controls (incorporating only the treatment). The plants were kept in a greenhouse with drip irrigation and anti-weed mesh for five months. At the end of the experiment, the plants were removed and two transverse sections of each inoculated stem, between the grafting point and the root crown, were prepared and sectioned longitudinally. The effects of the inoculated fungi were evaluated by measuring the lengths of the longitudinal vascular necroses in each direction from the point of inoculation and comparing them with those observed in the controls. For each inoculation point, a single necrosis length value was obtained by averaging the four necrosis length measurements/wound (upper left, bottom left, upper right, and bottom right). Finally, the two mentioned fungi were re-isolated from the measured lesions and morphologically identified to fulfill Koch’s postulates.

### 2.11. Statistical Analyses

The results of the in vitro mycelium growth inhibition assays were statistically analyzed using one-way analysis of variance (ANOVA), which was followed by a post hoc comparison of means using the Tukey test at *p* < 0.05 given that the homogeneity and homoscedasticity requirements were met according to the Shapiro–Wilk and Levene tests. For the in planta bioassays, in which the normality and homoscedasticity requirements were not met, the Kruskal–Wallis nonparametric test was used instead, with the Conover–Iman test for post hoc multiple pairwise comparisons. R statistical software was used for all of the statistical analyses [35].

## 3. Results

### 3.1. Nanocarriers Characterization

#### 3.1.1. Elemental Analysis

Based on the results obtained from the elemental analysis (Table 1), the content of MA in the g-C_3_N_4_-MA-COS NCs would be in reasonable agreement with what may be expected for a 0.5:0.5:1 ratio. As for the changes in elemental composition after the encapsulation of the *R. tinctorum* extract, taking into consideration that its main phytoconstituents are members of the anthraquinone family, with a C_14_H_8_O_2_ empirical formula, the amount of the extract may be close to 14 wt%.

#### 3.1.2. Vibrational Characterization

**Figure 1:**
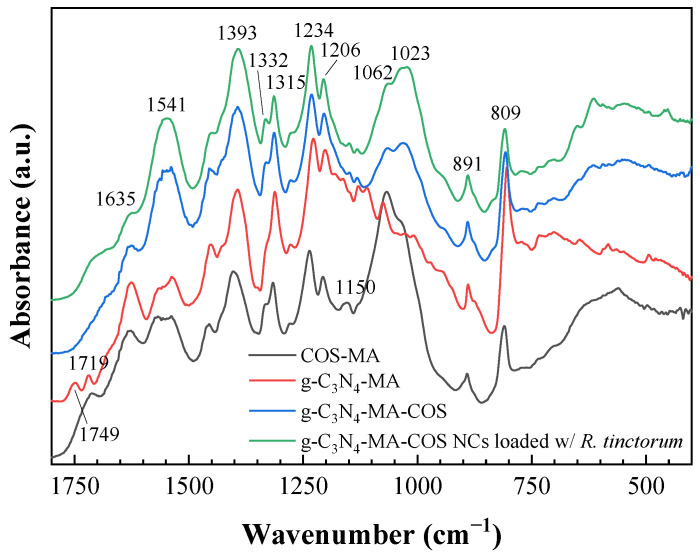
Fingerprint region of the infrared spectra of methacrylated chitosan oligomers (COS-MA), methacrylated graphitic carbon nitride (g-C_3_N_4_-MA), g-C_3_N_4_-MA-COS nanocarriers, and g-C_3_N_4_-MA-COS nanocarriers loaded with *R. tinctorum* extract.

The infrared spectrum of the g-C_3_N_4_-MA-COS nanocarriers showed absorption bands at 3173, 3050, 2924, 2364, 1635, 1541, 1393, 1332, 1315, 1234, 1206, 1150, 1062, 1023, 891, 809, 702, 614, 522, and 452 cm^−1^ (Figure 1). The band at 3170 cm^−1^ corresponds to the amine groups, the band at 3050 cm^−1^ is due to the C–H stretching vibration, the peak at 1393 cm^−1^ represents the bending vibration for CH_3_ groups, the band at 1062 cm^−1^ can be assigned to CH_2_ wagging from the CH_3_ groups, and the band at 698 cm^−1^ can be assigned to C-C bending. Characteristic absorption bands for chitosan appear at 2364 cm^−1^ (C-N asymmetric band stretching) and at 1023 and 1150 cm^−1^ (amine C-N stretching). Since both the chitosan and methacrylate groups display alkyl C-H stretching at 2924 cm^−1^, the increase in the intensity of this absorption band evidences the methacrylation [38]. The band at 1541 cm^−1^ arises from alkenyl C=C stretching, while the presence of the amide C=O stretching band at 1635 cm^−1^ supports methacrylation. The peaks at 1332, 1315, 1234, 1206, 891, and 809 cm^−1^ are characteristic of g-C_3_N_4_ [39], with the absorption bands at 1332 cm^−1^ and 1234 cm^−1^ being due to C–N stretching vibration [40], and those at 1315 and 809 cm^−1^ are due to triazine rings [41]. It should be noted that the infrared spectrum of the nanocarriers differs from that of the g-C_3_N_4_-MA precursor by the loss of resolution of the bands, which the latter one exhibits at 1749 and 1719 cm^−1^. Further, a comparison of FTIR spectra of the empty and *R. tinctorum*-loaded NCs exhibited no relevant spectral changes. 

#### 3.1.3. Morphology

**Figure 2:**
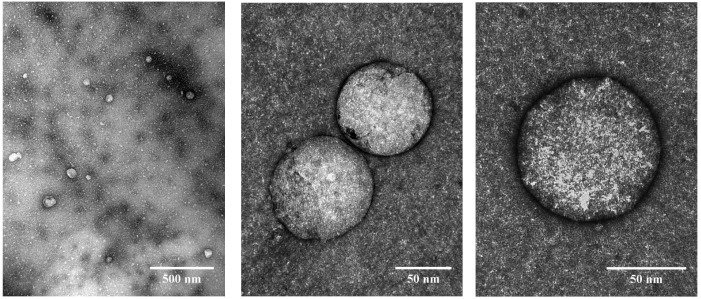
TEM micrographs of g-C_3_N_4_-MA-COS nanocarriers loaded with *R. tinctorum* extract.

The transmission electron microscopy images of the nanocarriers showed spherical nanoparticles consisting of an outer assembly of g-C_3_N_4_-MA-COS and a hollow space (Figure S1) where the phytochemicals can be accommodated, which in this case was the *R. tinctorum* extract (Figure 2). A histogram showing the size distribution is presented in Figure S2. The nanoparticles had a diameter of 85.5 ± 21.3 nm (mean ± SD), with a minimum size of 47 nm and a maximum size of 147 nm. The dispersity index value (0.25), which was lower than 0.3, and the existence of a single peak in the size distribution curve, suggest that the particles are monodisperse according to Sadeghi et al. [42].

#### 3.1.4. Thermal Analysis

**Figure 3:**
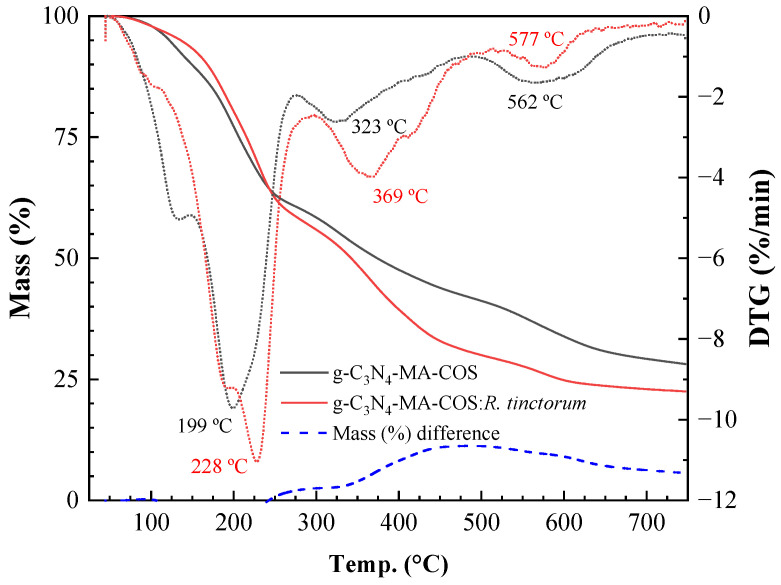
TG (left axis) and DTG (right axis) thermograms of g-C_3_N_4_-MA-COS NCs: empty NCs (black lines), NCs loaded with *R. tinctorum* extract (red lines).

The TG/DTG thermograms of the g-C_3_N_4_-MA-COS NCs, before and after encapsulation of *R. tinctorum* extract, are shown in Figure 3. Both of the samples showed three main effects at around 215, 346, and 570 °C. The former two effects can be attributed to the depolymerization of chitosan oligomers and decomposition of the substituted sites in the COS-MA moiety, respectively, while the third event may be ascribed to g-C_3_N_4_ deamination. The encapsulation of the *R. tinctorum* extract resulted in mass (%) differences that reached a maximum at ca. 500 °C, with a behavior similar to the one observed for lignin-MA-COS NCs loaded with the same extract [16]. Concerning the final residue at 750 °C, as expected, it was higher in the empty NCs (28.2%) than it was in the filled NCs (22.5%), and should be mainly attributed to char from COS [43]. 

### 3.2. Photocatalytic Activity

#### 3.2.1. Rhodamine B Degradation

**Figure 4:**
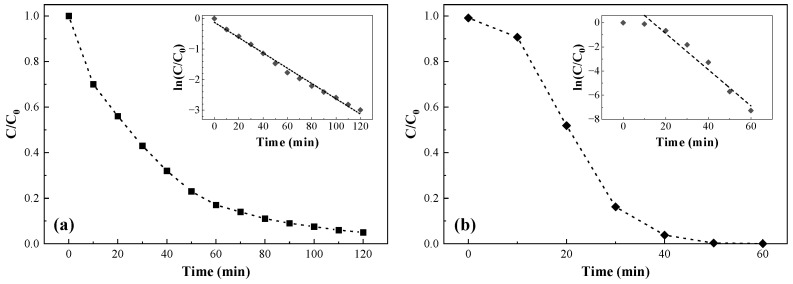
Photocatalytic activity for (**a**) RhB degradation and (**b**) U(VI) reduction with g-C_3_N_4_-MA-COS NCs at pH 6 under visible light irradiation.

As expected from the presence of the g-C_3_N_4_ moiety, the g-C_3_N_4_-MA-COS nanoparticles exhibited photocatalytic activity under visible light irradiation, and they were able to degrade RhB (while its self-degradation, which is not shown in Figure 4a, was negligible). Under the experimental conditions (pH 6), the efficiency of RhB degradation reached 90% after 90 min of illumination. The dye degradation fitted to the pseudo-first-order kinetics had a calculated degradation constant (*k*) of 0.025 min^−1^ (R^2^ = 0.9924).

#### 3.2.2. Uranium(VI) Reduction

Firstly, the adsorption properties of the NCs for U(VI) were investigated in the dark, and a low removal rate (<5%) over a 24 h period was found. In contrast, a substantial reduction of U(VI) under visible light irradiation was observed, reaching complete removal after 50 min (Figure 4b), with a pseudo-first-order kinetics constant *k* = 0.15 min^−1^ (R^2^ = 0.964).

### 3.3. Encapsulation and Release Efficiencies

An almost complete encapsulation (EE = 95–97%) of the *R. tinctorum* extract was achieved. However, the release efficiency upon the enzymatic degradation of the NCs using a commercial chitosanase (EC 3.2.1.132) was substantially lower, with RE values that were in the 74–81% range.

### 3.4. Antimicrobial Activity

#### 3.4.1. Antibacterial Activity

The g-C_3_N_4_-MA-COS nanocarriers, in the absence of a bioactive compound inside, inhibited the growth of *X. ampelinus* (the causal agent of bacterial necrosis of grapevines) at a concentration of 1500 μg·mL^−1^, i.e., at the highest concentration tested, while the NCs loaded with *R. tinctorum* extract presented over 10 times more germicide activity, fully inhibiting the phytopathogen at a dose as low as 125 μg·mL^−1^ (Table 2).

#### 3.4.2. Antifungal Activity

**Figure 5:**
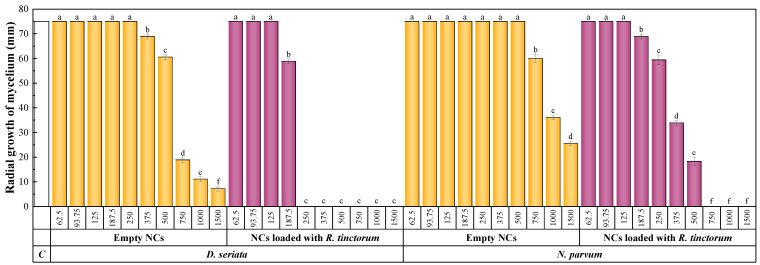
Diameter (in mm) of the mycelial growth of *D. seriata* and *N. parvum* for different concentrations of the *g*C_3_N_4_-MA-COS nanocarriers, before and after the encapsulation of *R. tinctorum* extract. The letters above concentrations indicate that they are not significantly different at *p* < 0.05. ‘C’ and ‘NCs’ stand for control and nanocarriers, respectively.

Figure 5 shows the radial growth of the *D. seriata* and *N. parvum* colonies for the g-C_3_N_4_-MA-COS NCs, before and after the encapsulation of *R. tinctorum* extract. It could be observed that, in the absence of a bioactive compound, the NCs failed to fully inhibit mycelial growth in either of the phytopathogens studied. However, when they were loaded with an extract of *R. tinctorum*, the full inhibition of mycelial growth was achieved at a concentration of 250 and 750 µg·mL^−1^ for *D. seriata* and *N. parvum*, respectively.

#### 3.4.3. In Planta Bioassays

**Figure 6:**
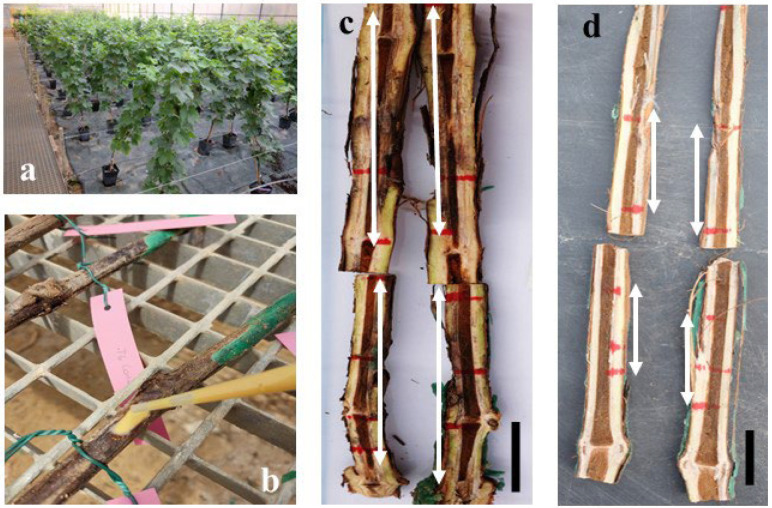
(**a**) General view of the in planta assay; (**b**) application of the *R. tinctorum* extract-loaded NCs on one of the wounds made on a young ‘Tempranillo’ grapevine plant; (**c**) necrosis lengths in control plants; (**d**) necrosis lengths in treated plants. Bars = 1 cm. Necrosis lengths are indicated with arrows to facilitate identification.

As a first step towards field application, the NCs were assayed as a wound protection treatment against two fungal pathogens of the family *Botryosphaeriaceae* associated with grapevine black dead arm disease in greenhouse conditions (Figure 6). The MIC values determined in the previous section were chosen as the application dose (i.e., 250 and 750 µg·mL^−1^ for *D. seriata* and *N. parvum*, respectively). Statistically significant differences were found in the longitudinal vascular necroses between the plants that were treated with the *g*-C_3_N_4_-MA-COS NCs loaded with the *R. tinctorum* hydromethanolic extract and the positive controls (Table 3), confirming that they had some protective effect against both of the trunk pathogens. No phytotoxicity symptoms were observed in the negative controls.

## 4. Discussion

### 4.1. On the Nanocarriers Structure and the Encapsulation Mechanism

**Figure 7:**
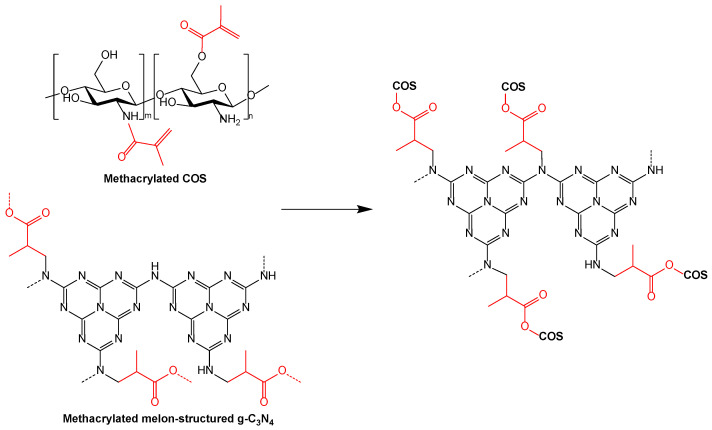
Hypothetical assembly of the methacrylated COS and methacrylated g-C_3_N_4_ moieties.

The characterization results, in particular, the TEM micrographs, evidenced the g-C_3_N_4_-MA-COS assembly as being typical of nanocarriers. Nevertheless, the binding of their moieties remains an unsolved problem. It can be hypothesized that it is the result of grafting one of the moieties onto the other (Figure 7), but a polymeric scaffold cannot be ruled out.

Concerning the nature of the encapsulation of *R. tinctorum* phytochemicals in g-C_3_N_4_-MA-COS NCs, one would have to consider whether it would be based on physical or chemical entrapment. In the case of physical entrapment, no changes or minimal changes in the vibrational spectrum compared to the parent compounds would be expected, whereas in chemical entrapment, spectral changes might occur due to a possible chemical interaction between the extract components and the NCs [44]. Given that the encapsulation of *R. tinctorum* in the NCs resulted in no shifts of the characteristic bands of the FTIR spectra, thus suggesting that there was no modification or interaction between the ‘shell’ assembly and the phytochemicals, it may be safely assumed that the extract was physically entrapped (encapsulated) within the NCs, which is in agreement with other works [45,46].

### 4.2. Photocatalytic Activity

Concerning RhB degradation, the pseudo-first-order kinetics constant obtained in the experiment (*k* = 0.025 min^−1^) was about 3.8 times higher than that which was reported for the reduced graphene oxide/chitosan composite aerogels supported g-C_3_N_4_ photocatalyst (*k* = 0.0065 min^−1^) [47] and it was comparable to the value reported by Xu et al. [48] for a chitosan/TiO_2_@g-C_3_N_4_ nanocomposite membrane (0.0238 min^−1^), although it should be noted that operative conditions were different (30 mg·L^−1^ RhB, pH = 2, and a 30 W LED lamp in the former study, and 5 mg·L^−1^ RhB and 100 W LED lamp in the latter one), so comparisons should be made with caution. If a comparison with pristine (non-doped) g-C_3_N_4_ was made, the reported *k* value would be intermediate between those of g-C_3_N_4_ and high-surface-area porous g-C_3_N_4_ (0.014 and 0.131 min^−1^, respectively) reported by Dong and Zhang [31]. It is worth noting that the indicated degradation rate would also be highly influenced by the pH value (six in our case for consistency reasons, provided that it is the usual choice in U(VI) reduction experiments) given that it substantially increases with the decrease in the pH values [49].

As for uranium(VI) reduction, no examples of composites consisting of g-C_3_N_4_ and chitosan have been reported in the literature, so a comparison with g-C_3_N_4_-only experiments was provided instead. The removal rate (*k* = 0.15 min^−1^) was intermediate between those of the bulk pristine g-C_3_N_4_ (0.04/0.06 min^−1^) and the highly mesoporous g-C_3_N_4_ samples (0.27 min^−1^) prepared using silica NPs (~12 nm) as a template [32,50] and it was comparable to other values obtained for pristine g-C_3_N_4_ and different g-C_3_N_4_/co-catalyst systems, although *k* values in the 0.05–0.42 min^−1^ range have been reported [51]. Again, although the pH was the same in all of the aforementioned studies, different initial concentrations of U(VI) and photocatalyst:U(VI) ratios were employed, so comparisons are made merely by way of guidance.

### 4.3. On the Antimicrobial Activity

With regard to the antibacterial activity, to the best of the authors’ knowledge, no data on the efficacy of the *R. tinctorum* extract against *X. ampelinus* are available. Nonetheless, previous studies in which the bactericidal activity of COS has been evaluated against the same isolate showed that the bacterial growth was completely inhibited at 1500 µg·mL^−1^ [52,53,54,55], which is a similar MIC to the one that was obtained in this work for the empty g-C_3_N_4_-MA-COS NCs.

As for the antifungal activity, a comparison of the EC_50_ and EC_90_ concentrations (i.e., the concentrations at which mycelial growth was inhibited by 50 and 90%, respectively) obtained for the *R. tinctorum*-loaded NCs versus those obtained for the unencapsulated *R. tinctorum* extract, which was previously reported in [26] against the same fungal isolates, is presented in Table 4. If the actual bioactive product weight is considered (ca. 14 wt%, according to elemental analysis results), the corrected EC_50_ and EC_90_ values would be noticeably lower than those that were obtained with the unencapsulated *R. tinctorum* extract. Although such a finding would support the hypothesis that the controlled release properties of the NCs would minimize wastage and leaching of the active ingredients, it should be taken as a first approximation given that the contribution of COS to the antifungal activity has been discarded (a simplification supported by its noticeably higher EC_90_ values, viz. 1180 and 1327 µg·mL^−1^ against *D. seriata* and *N. parvum*, respectively [56]).

Regarding the in vivo results, it should be noted that the protective effect was limited by the assayed dose (i.e., the previously in vitro-determined MICs were 250 and 750 μg·mL^−1^ for *D. seriata* and *N. parvum*, respectively), which suggests that a higher concentration would be needed to achieve full protection (in terms of reduction of vascular necrosis) in the field tests against the two pathogens.

### 4.4. Mechanism of Action

With regard to the photocatalytic activity, it should be only ascribed to the g-C_3_N_4_ moiety. In the case of RhB degradation, the photocatalytic pathway includes N-de-ethylation, chromophore structure cleavage, ring-opening, and mineralization processes [57]. Although hydroxyl radicals (^•^OH) generated via the electron-induced multistep reduction of O_2_ (induced by the light irradiation of g-C_3_N_4_) have generally been considered to be the main reactive oxidation species [58], more recent works have suggested that photogenerated superoxide (O_2_^•^ or HOO^•^) species would play a major role in the photocatalytic process [57]. 

In relation to the U(VI) photocatalytic reduction mechanism, it is not fully understood, but Zhang et al. [51], based on a thorough review of the state-of-the-art, suggested two possible mechanisms:

(1) $${\mathrm{UO}}_{2}^{2+}+e^{-}\to {\mathrm{UO}}_{2}^{+},\;{\mathrm{UO}}_{2}^{+}+e^{-}\to U^{4+},$$

(2) $${\mathrm{UO}}_{2}^{2+}+4H^{+}+2e^{-}\to U^{4+}+2H_{2}O,$$

with an associated redox potential (vs. NHE) that was in the +0.062 to +0.31 range in the case of Equation (1) (single photogenerated electron reduction process) and between +0.267 and +0.57 in the case of Equation (2) (in the presence of acidity and two photogenerated electrons).

Concerning the antimicrobial applications, it should be noted that antimicrobial activity has been reported for both g-C_3_N_4_ and COS. In the case of g-C_3_N_4_, it is ascribed to photocatalytic reactive oxygen species (ROS) generation [59], while in the case of COS, different mechanisms of action have been proposed [60], including the interaction of the positively charged COS with negatively charged phospholipid components (which results in increased membrane permeability and the leakage of cellular contents), the deprivation to fungi of trace elements that are essential for normal growth due to COS chelating action, and the inhibition of mRNA synthesis due to its binding to fungal DNA (which affects protein and enzyme production). However, as shown in the in vitro studies, the effectiveness of the empty NCs against the assayed pathogens would be very limited as they have high MIC values. Thus, the influence of the g-C_3_N_4_-MA-COS shell on the antimicrobial activity may be regarded as being negligible in comparison to that of the encapsulated product, and the role of the NCs in these potential applications would be mainly restricted to the targeted delivery of the bioactive products.

In this regard, in a similar fashion to previously reported lignin-based NCs, which successfully inhibited the growth of ligninolytic enzymes-producing microorganisms by releasing encapsulated fungicides [15], the presence of the COS moiety in the NCs studied herein may be useful to selectively release the encapsulated bioproducts (in this case, *R. tinctorum* extract) against bacterial and fungal pathogens. Such a release would be triggered upon the interaction with the chitosanase produced by the microorganisms, which would degrade the g-C_3_N_4_-MA-COS shell and selectively release its content. For instance, in the case of *N. parvum*, the chitosanolytic activity of the fungus is well established, and endo-chitosanase (GH75, R1GTL6) has been shown to have a central role in the host infection mechanism [61]. It should also be noted that, albeit they are less specific and have a lower efficiency, chitinases and cellulases also break down chitosan [62], and these enzymes are widely present in *Botryosphaeriaceae* and other wood-degrading fungi [63], suggesting that there is a wide applicability of the NC-based treatments.

Finally, concerning the *R. tinctorum* antimicrobial activity, it may be ascribed to a combined effect of both the anthraquinones and phenols present in the extract [26].

### 4.5. Limitations of the Study and Prospects of Nanocarriers for Agrochemical Delivery

The data presented herein support the rationale that the nanocarriers consisting of naturally or synthetically sourced polymers and inorganic materials can enhance the stability and performance of a broad range of active ingredients, which is in agreement with Pinto et al. [3]. However, despite their promising efficacy, the questions of economy and scalability must still be addressed in subsequent studies to determine whether these NCs may be scalable and transformable into economically viable solutions to solve present and future agricultural problems (i.e., technical constraints concerning the production of NCs for use in agriculture should be correlated with the economical boundaries which limit the production costs and configure the potential revenues for the producers [2]). Further, the issues related to safety assessment, evaluation standards, registration policies, and public concern should also be addressed, as noted by An et al. [64].

Concerning the NCs’ prospective practical use, it is worth noting that the current level of knowledge does not yet allow a fair and unbiased assessment of the pros and cons that will arise from the use of nano-based systems for the encapsulation of bioactive products and their use in agriculture. Nonetheless, one may be optimistic about the applicability of NCs such as the ones presented in this work given that at the moment there are already over 200 products commercialized worldwide that can be classified as nano-based products in agriculture and that the nanotechnology market in the agricultural sector is expected to grow at a compound annual growth rate of ca. 28% [4].

## 5. Conclusions

In this work, methacrylate anhydride was shown to be an efficient cross-linking agent to bind g-C_3_N_4_ and COS in a weight ratio of 0.5:1, leading to the formation of monodisperse nanocarriers with a mean size of 85.5 ± 21.3 nm. Regardless of whether the g-C_3_N_4_-MA-COS assembly is considered to be the result of grafting one of its moieties onto the other or a polymeric scaffold, the photocatalytic activity and the behavior as carriers for the targeted delivery of bioactive products have been demonstrated. Due to the presence of the g-C_3_N_4_ moiety, the nanocarriers were able to efficiently photodegrade RhB and photoreduce U(VI) with rate constants of 0.025 and 0.15 min^−1^, respectively, which are higher than those of bulk pristine g-C_3_N_4_. In turn, the presence of the COS moiety allowed a selective release of encapsulated *R. tinctorum* extract against certain grapevine phytopathogens. The in vitro results supported the more efficient use of the bioactive product, with MIC values that are noticeably lower than those which were obtained with the unencapsulated extract. Moreover, the application of the extract-loaded NCs as a pruning wound protection product in young ‘Tempranillo’ grapevine plants that were artificially infected with *N. parvum* and *D. seriata* resulted in a significant reduction in the necrosis lengths. Therefore, g-C_3_N_4_-MA-COS nanocarriers may hold promise as a multifunctional platform as they are more versatile than conventional chitosan-based nanoparticles are for environmental and agrochemical delivery applications.

## 6. Patents

The work reported in this manuscript is related to the Spanish patent with application number P202230668 (‘Nanomaterial basado en el autoensamblaje de g-C_3_N_4_ y oligómeros de quitosano, proceso de obtención y usos’ which translates as ‘Nanomaterial based on self-assembly of g-C_3_N_4_ and chitosan oligomers, obtaining process and uses’ (tr.)), which was filed on 20 July 2022.

## Figures and Tables

**Table 1:** EDX elemental analysis results.

Samples	Elemental Composition (%)				Ref.
	C	H	N	O	
Methacrylic acid	55.7	7.0	-	37.3	-
COS	42.9	6.3	6.8	44.0	[36]
g-C_3_N_4_ (g-C_3_N_4_ – g-C_3_N_4.2_ range)	39.4 (32.6–46.1)	10.1 (0–20.3)	50.4 (47.1–53.8)	-	[37]
Methacrylated COS	48.8 (45.5–52.1)	6.5 (5.2–7.8)	3.8 (1.1–6.5)	40.9 (39.0–42.8)	[36]
Methacrylated g-C_3_N_4_	43.7	-	40.8	15.5	This work
g-C_3_N_4_-MA-COS assembly	47.1	-	16.1	36.8	This work
g-C_3_N_4_-MA-COS:*R. tinctorum*	52.1	-	16.1	31.8	This work

**Table 2:** Antibacterial activity of the g-C_3_N_4_-MA-COS nanocarriers (NCs), before and after the encapsulation of *R. tinctorum* extract, against the phytopathogen *X. ampelinus*.

Treatment	Concentration (µg·mL^−1^)									
	62.5	93.75	125	187.5	250	375	500	750	1000	1500
Empty NCs	+	+	+	+	+	+	+	+	+	−
NCs loaded with *R. tinctorum*	+	+	−	−	−	−	−	−	−	−

“+” and “−“ indicate the presence and absence of bacterial growth, respectively.

**Table 3:** Results of the Kruskal–Wallis tests followed by multiple pairwise comparisons using the Conover–Iman procedure performed on the necrosis lengths caused by *D. seriata* and *N. parvum*. The mean of ranks values accompanied by the same letters are not significantly different (*p*-value (one-tailed) < 0.047 and 0.031 for *D. seriata* and *N. parvum*, respectively; α = 0.05).

Treatment	*D. seriata*			*N. parvum*		
	Mean of Ranks	Groups		Mean of Ranks	Groups	
NCs-*R. tinctorum*	30.750	A		30.607	A	
Control	44.750		B	45.750		B

**Table 4:** Comparison between the effective concentrations (EC_50_ and EC_90_, in µg·mL^−1^) against *D. seriata* and *N. parvum* of the g-C_3_N_4_-MA-COS nanocarriers loaded with *R. tinctorum* extract and those of the equivalent encapsulated and non-encapsulated *R. tinctorum* extracts.

Pathogen	Effective Concentration	g-C_3_N_4_-MA-COS- *R. tinctorum*	Encapsulated *R. tinctorum* (ca. 14 wt%)	*R. tinctorum* Extract [26]
*D. seriata*	EC_50_	193.1	27.0	78.0
	EC_90_	241.0	33.7	87.8
*N. parvum*	EC_50_	362.7	50.8	92.3
	EC_90_	631.5	88.4	184.0

## Data Availability

The data presented in this study are available on request from the corresponding author. The data are not publicly available due to their relevance to an ongoing Ph.D. thesis.

## Supplementary Materials

The following supporting information can be downloaded at: https://www.mdpi.com/article/10.3390/ma15248981/s1, Figure S1. TEM micrographs of empty g-C_3_N_4_-MA-COS nanocarriers without negative staining (upper left) and with negative staining (other five images) at different magnifications; Figure S2. Histogram of the nanocarrier size distribution.
