# Supplementary material for: Multifunctional Nanocarriers Based on Chitosan Oligomers and Graphitic Carbon Nitride Assembly
Source: Materials (Basel). 2022 Dec 15;15(24):8981. doi: 10.3390/ma15248981 (PMC9785438; doi:10.3390/ma15248981)
Supplement: Supplementary file 1 [file materials-15-08981-s001.zip › materials-2012891-supplementary.pdf]

Multifunctional nanocarriers based on chitosan oligomers and graphitic carbon nitride assembly

A. Santiago-Aliste, E. Sánchez-Hernández, N. Langa-Lomba, V. González-García,
J. Casanova-Gascón, J. Martín-Gil and P. Martín-Ramos

SUPPLEMENTARY MATERIAL

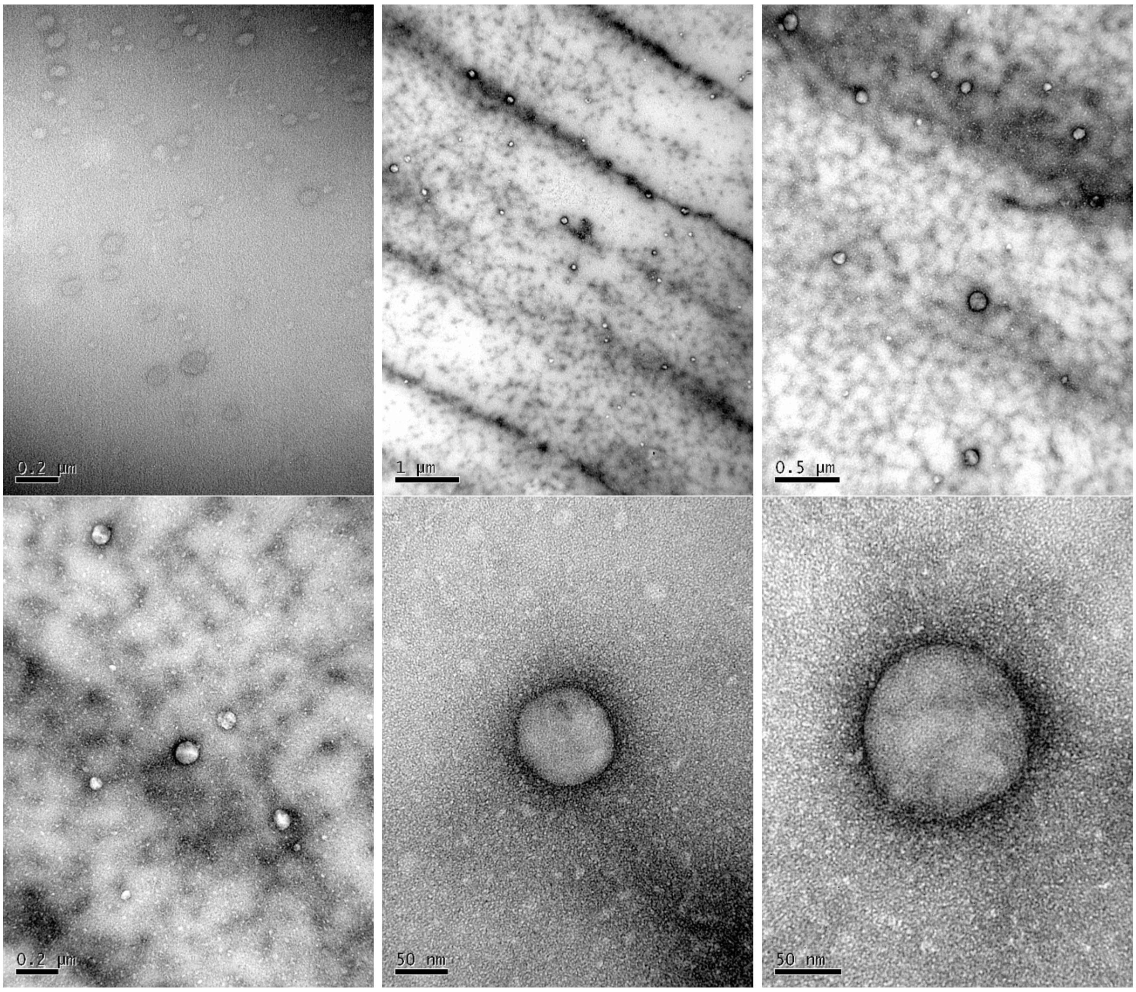

Figure S1. TEM micrographs of empty g-C_3N_4-MA-COS nanocarriers without negative staining (upper left) and with negative staining (other five images) at different magnifications.

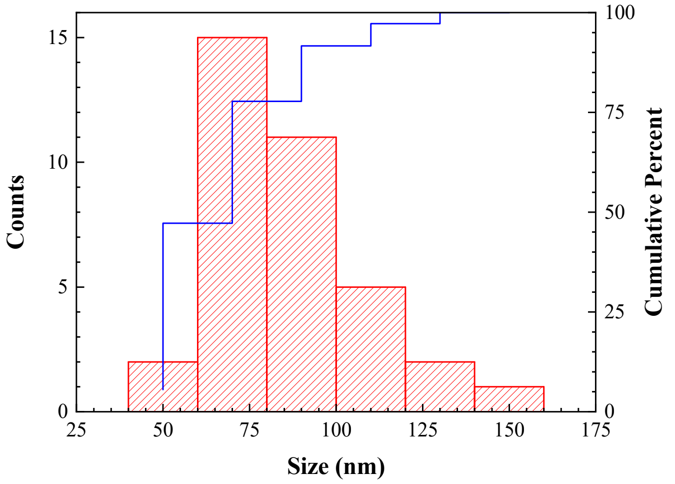

Figure S2. Histogram of the nanocarrier size distribution.
